# Profiles of Childhood Maltreatment and Defenses: Associations with Personality Functioning in Emerging Adulthood

**DOI:** 10.1007/s40653-025-00710-4

**Published:** 2025-05-06

**Authors:** Jacopo Tracchegiani, Andrea Fontana, Ilaria Maria Antonietta Benzi, Laura Muzi, Nicola Carone

**Affiliations:** 1 Department of Brain and Behavioral Sciences, University of Pavia, Piazza Botta 11, 27100 Pavia, Italy; 2 Department of Human Science, LUMSA University, Piazza delle Vaschette 101, 00193 Rome, Italy; 3 Department of Psychology, University of Milan–Bicocca, Piazza dell’Ateneo Nuovo, 1, 20126 Milan, Italy; 4 Department of Philosophy, Social Sciences, Humanities and Education, University of Perugia, Piazza G. Ermini 1, 06123 Perugia, Italy; 5 Department of Systems Medicine, University of Rome Tor Vergata, Via Montpellier 1, 00133 Rome, Italy

**Keywords:** Personality functioning, AMPD, Childhood maltreatment, Defenses, Emerging adulthood

## Abstract

Childhood maltreatment and maladaptive emotion regulation processes are two interrelated risk factors for impaired personality functioning in emerging adults. However, the impact of the co-occurrence of different childhood maltreatment experiences and maladaptive defensive functioning on personality functioning remains underexplored. This study aimed to identify distinct profiles of maltreatment and defenses while examining their association with self- and interpersonal personality functioning impairments. A community sample of 1,315 cisgender emerging adults (*M*_*age*_ = 24.33, *SD* = 2.75; 75.06% assigned female at birth; 76.43% heterosexual) completed the Childhood Trauma Questionnaire-Short Form (CTQ-SF), the Defense Mechanisms Rating Scales–Self-Report-30 (DMRS-SR-30), and the Level of Personality Functioning Scale-Brief Form (LPFS-BF). Latent profile analysis suggested two profiles: *High-Trauma/Maladaptive Defenses* (HT/MD) and *Low-Trauma/Adaptive Defenses* (LT/AD). The first profile was characterized by higher exposure to childhood maltreatment and greater reliance on maladaptive defenses, while the second profile exhibited lower maltreatment exposure and greater reliance on adaptive defenses. Additionally, individuals in the HT/MD profile reported significantly greater impairments in self- and interpersonal personality functioning compared to those in the LT/AD group. These findings suggest that co-occurrence of maltreatment is linked to higher maladaptive defenses, underscoring their impact on personality functioning impairments. Clinically, interventions targeting defensive functioning may help maltreated emerging adults develop healthier self- and interpersonal functioning, facilitating their adaptation to adulthood.

## Introduction

In recent decades, the transition from adolescence to adulthood has become known as the distinct developmental stage of *emerging adulthood* (18–29 years). Arnett (2000) proposed five pillars, or domains, of this phase: (1) identity exploration, which involves the exploration of various possibilities in love, work, and worldviews to form a coherent sense of identity; (2) instability, due to numerous life transitions (with respect to, e.g., residence, relationships, education, and work); (3) self-focus, concerning the fewer social obligations and responsibilities towards others, allowing for a focus on personal development; (4) the feeling of being in-between, capturing the subjective experience of being neither fully adolescent nor fully adult, but in a transitional phase; and (5) possibilities, reflecting the sense of having numerous options for the future and a belief that one can achieve personal goals.

The multiple developmental challenges in identity and interpersonal domains that characterize emerging adulthood make this stage a critical juncture for the development of psychopathology. Indeed, many symptoms and disorders become diagnosable during this period (e.g., Solmi et al., 2021), particularly with respect to anxiety, depression, substance use, and other externalizing symptoms (Persike et al., 2020). Emerging adulthood is also a vulnerable period for the onset of dysfunctional personality traits and disorders (Wright et al., 2011). Although maladaptive personality traits may increase during childhood, personality disorders typically emerge during the transition between adolescence and adulthood, when individuals must become emotionally, socially, and cognitively prepared to integrate knowledge and experiences about themselves and others, and subsequently assume adult rights, responsibilities, and social roles (Chanen & Thompson, 2019).

Research and clinical practice are on the brink of a paradigm shift from categorical to more dimensional models of personality and related disorders, as exemplified by the publication of the *Alternative DSM-5 Model for Personality Disorders* (AMPD) (APA, 2013; Skodol et al., 2015). The AMPD assesses personality functioning severity (Criterion A) and maladaptive traits (Criterion B). Of note, Criterion A encompasses both *self-impairment*, including identity issues (e.g., difficulty maintaining the boundary between self and other, unstable self-esteem, inadequate self-regulation) and self-direction (e.g., inability to develop and pursue meaningful goals or follow personal and cultural standards of prosocial behavior); as well as *interpersonal impairment*, which comprises difficulties with empathy (e.g., difficulty understanding and respecting others’ experiences and motivations, inaccurate perception of the effects of one’s behavior on others) and intimacy (e.g., unstable, brief, or enmeshed social connections, reduced or excessive desire and capacity for closeness) (Bender et al., 2011).

The development of the AMPD model drew from existing psychodynamic frameworks, demonstrating notable conceptual affinities (e.g., Kampe et al., 2018; Yalch, 2020). For example, the focus on self- and interpersonal functioning in the AMPD Criterion A aligns with identity and object relations in the Object Relations Theory (Clarkin et al., 2020). Despite AMPD’s recognized value in advancing a dimensional perspective on personality, particularly through its assessment of personality functioning domains, debate remains regarding its suitability as an official diagnostic system. In particular, drawing from Object Relations Theory, certain aspects not directly addressed by AMPD—such as defenses—could contribute to a more comprehensive understanding of personality functioning impairment (Clarkin et al., 2020; Roche et al., 2018).

Such impairment has been linked to a wide range of dysfunctional symptoms and/or disorders, including depression (Vittengl et al., 2023), post-traumatic stress disorder (Møller et al., 2021), and eating disorders (Klein et al., 2022) in adults, as well as overall psychopathology in youth (Iannattone et al., 2024). However, there is limited understanding of AMPD-based personality functioning in emerging adulthood and the factors influencing identity and interpersonal domains (i.e., Criterion A) during this period.

Abusive or neglectful experiences prior to the age of 18 years represent significant risk factors for developmental task failure, hindering subsequent adaptation (Cicchetti & Banny, 2014). In particular, such experiences may lead to maladaptive self-regulation and distorted self and other representations (Doyle & Cicchetti, 2017; Handley et al., 2019). Consequently, emerging adults with a history of childhood maltreatment may lack the necessary resources to navigate this life stage and stand at increased risk of developing psychopathology (Caspi et al., 2020). The detrimental impact of childhood maltreatment on self and other representations is likely to persist throughout life (Back et al., 2020; Gander et al., 2020; Hecht et al., 2014; Stone et al., 2023). Thus, an adverse childhood environment may exacerbate developmental challenges related to personality functioning, such as emotion regulation, self-image integration, and stable relationship formation (Cicchetti, 2016), with higher maltreatment severity correlated with greater impairment (Freier et al., 2022).

Research has shown that maltreatment often leads to maladaptive self-regulation in emerging adults (Warmingham et al.,^2022^), suggesting that emotion regulation strategies in maltreated individuals are strictly linked to their childhood experiences. The literature identifies two primary processes of emotion regulation: explicit and implicit (Gyurak et al., 2011). Within implicit processes, defenses “can alter perception of any or all of the following: subject (self), object (other person), and idea or feeling (affect)” (Vaillant, 2020 p. 1025). These mechanisms protect an individual’s stability and integrity in response to traumatic or high-stress events (Cramer, 2015a), and are embedded in the personality structure (e.g., Kernberg, 1984; Lingiardi & McWilliams, 2017) and shaped by early experiences (Carone et al., 2023; Prunas et al., 2019). Unlike voluntary, situation-based coping strategies, defenses are largely automatic, unintentional, and dispositional, meaning they are independent of immediate situational demands and are organized hierarchically within an individual’s personality (Cramer, 2015a, b).

According to the hierarchal defense model which framed this study (Perry, 1990; Vaillant, 1977), defenses range from maladaptive (e.g., acting out, passive aggression) to highly adaptive forms (e.g., self-observation, affiliation), with the latter considered more developmentally mature. For example, the use of defenses is universal, and therefore not inherently psychopathological. However, excessive and inflexible reliance on maladaptive defenses may lead to personality disorders, whereas greater use of adaptive defenses protects against psychopathology (Cramer, 1999; Vaillant, 1977). Notably, given that the same individual may rely on different defensive levels, it is relevant that the absence of mature defenses—rather than the simple presence of immature—may be particularly relevant in explaining severe personality impairments (McWilliams, 2011).

From this perspective, childhood maltreatment may hinder the development of adaptive defensive styles, making immature defenses more accessible and likely to be used during stressful situations (Cramer, 2015a). However, it remains unclear whether some forms of childhood maltreatment are more related with defenses, as most research has focused on isolated associations between distinct forms of trauma and specific defenses (e.g., Finzi-Dottan & Karu, 2006). So doing, previous research has overlooked the co-occurrence of multiple maltreatment experiences and the simultaneous presence of different defensive processes within the same individual. Furthermore, in individuals who experienced childhood maltreatment, frequent and pervasive use of immature and/or neurotic defenses at the expense of mature defenses may contribute to more severe impairment in overall personality functioning, encompassing self and interpersonal domains (McWilliams, 2011; Perry et al., 2013).

Given that maltreatment experiences often co-occur (Debowska et al., 2017), are associated with diverse maladaptive defenses which may manifest simultaneously across individuals (e.g., Ferrajão et al., 2023; Mahmoudvand et al., 2024), and are linked to impairments in personality functioning (e.g., Back et al., 2020; Gander et al., 2020), it is plausible that distinct profiles of childhood maltreatment experiences and defenses may have different implications for personality functioning.

### Childhood Maltreatment and Defenses

Around 400 million children under 5 years of age regularly maltreated at the hands of parents and caregivers (UNICEF, 2024). Child maltreatment includes acts of commission (i.e., physical, emotional, and sexual abuse) and/or omission (i.e., physical and emotional neglect) that fail to provide protection, induce uncontrollable fear, and cause or threaten harm to a child (Leeb et al., 2008). Emotional abuse, representing one of the most prevalent forms of maltreatment (Stoltenborgh et al., 2015), has been shown to be more significantly associated with psychopathology and maladaptive personality patterns than other forms (Baldwin et al., 2023; Lobbestael et al., 2010). However, childhood maltreatment rarely occurs in isolation (Debowska et al., 2017)—rather, multiple forms of abuse and neglect often co-occur, with greater cumulative exposure increasing the likelihood of developing personality disorders (Dong et al., 2004).

A growing body of research suggests that exposure to high-stress events, such as childhood maltreatment, often results in increased use of maladaptive defenses in both adults and emerging adults (Callahan & Hilsenroth, 2005; Carone & Tracchegiani, 2025; Finzi-Dottan & Karu, 2006). Over time, these defenses may become ingrained into the individual’s personality, persisting beyond their appropriate developmental stage (Cramer & Block, 1998). According to the hierarchal model of defense mechanisms (Perry, 1990; Vaillant, 1977), defenses are typically categorized into mature, neurotic, and immature. Immature defenses involve more significant distortion of one’s self, others, or external reality relative to more adaptive defenses. Neurotic defenses include fewer distortions of internal or external reality, excluding the emotional or cognitive component of experience from conscious awareness. For example, repression shields the self from awareness about a current or past intrapsychic conflict, leaving the individual to experience and express the affective component without recognizing what it is and its associated idea. Conversely, isolation of affect prevents an individual from fully experiencing negative affects tied to past or present intrapsychic conflicts while preserving cognitive awareness of the events. Mature defenses foster adaptive solutions, self-reflection, and increased awareness of internal and external stressors and their associated negative affects (Perry, 1990).

Empirical studies have found a positive and significant relationship between childhood maltreatment, both cumulative and distinct, and increased reliance on immature defenses in both clinical and non-clinical samples (Ferrajão et al., 2023; Finzi-Dottan & Karu, 2006; Mahmoudvand et al., 2024; Musetti et al., 2023; Wang et al., 2021). For example, adult victims of sexual and physical abuse are more likely to exhibit higher levels of immature defenses compared to non-abused or non-neglected control groups (Callahan & Hilsenroth, 2005; Finzi et al., 2003). Furthermore, in emerging adults, immature defenses have been shown to mediate the relationship between childhood emotional abuse and psychopathological symptoms (Finzi-Dottan & Karu, 2006). In addition to relying on immature defenses, research has shown that individuals exposed to childhood maltreatment are also more likely to exhibit a heightened reliance on neurotic defenses (Ferrajão et al., 2023; Mahmoudvand et al., 2024). Conversely, mature defenses may serve as protective factors, potentially mitigating the negative impact of childhood adversity on later mental health outcomes (Vaillant, 2003).

### Associations with Personality Functioning

Early experiences of childhood maltreatment play a critical role in the development of personality functioning. Meta-analyses have identified associations between childhood maltreatment and constructs operationalized within the AMPD Criterion A, such as mentalization (Benzi et al., 2023; Yang & Huang, 2024), self-esteem (Zhang et al., 2022), loneliness (de Heer et al., 2024), and empathy (Zhang et al., 2023). Two studies using the AMPD framework found relationships between the severity of impairment in personality functioning and all types of childhood maltreatment—especially emotional abuse in adolescence and physical neglect and emotional abuse in emerging adulthood (Back et al., 2020; Gander et al., 2020). One study analyzed specific associations with the four subcomponents of personality functioning (i.e., identity, self-direction, empathy, intimacy), finding that emotional abuse impacted all domains of personality functioning, while emotional neglect specifically affected intimacy (Gander et al., 2020). Furthermore, maltreatment has been found to be more strongly associated with impairment in self and interpersonal functioning than with specific maladaptive personality traits (i.e., Criterion B; Back et al., 2020). Nevertheless, most research has focused on personality traits rather than personality functioning (for a review, see Back et al., 2021).

Given the established relationship between maltreatment and the use of immature and neurotic defenses, individuals with a history of maltreatment may struggle to reconcile conflicting aspects of their abusive caregivers’ behaviors, leading to fragmented perceptions of others, their parents, and themselves. Consistent with a dimensional perspective on personality, studies have shown that neurotic and maladaptive defenses correlate positively with identity and interpersonal impairment, while adaptive defenses correlate negatively (Roche et al., 2018; Sarrar & Goth, 2022). This fragmentation may contribute to impaired representations of self and interpersonal difficulties. For instance, survivors of sexual abuse may see themselves as damaged and unlovable, reinforcing interpersonal patterns that hinder healthy social relationships (Callahan & Hilsenroth, 2005). Based on previous evidence, it is plausible that the co-occurrence of multiple maltreatment experiences alongside the simultaneous use of different defensive processes may have compounding detrimental effects on personality functioning.

### The Present Study

Given the complex interaction between childhood maltreatment experiences and defenses, it is likely that distinct trauma-defense profiles emerge, each with unique implications for personality functioning. While person-centered approaches, such as latent profile analysis (LPA), have been increasingly used in trauma research (Debowska et al., 2017), to the best of our knowledge no study has still applied this method to explore the relationship between maltreatment, defenses, and personality functioning. The focus on emerging adults is pertinent, as impairment in self and interpersonal personality functioning due to childhood maltreatment can severely impact developmental challenges during this life stage (e.g., identity exploration, increased self-focus, commitment and intimacy in relationships, responsibility in new social contexts; Arnett, 2000). Relatedly, emerging adulthood is a time in which individuals are expected to decrease their reliance on immature defenses (Vaillant, 1977).

The primary research aim was to explore distinct emerging adults’ profiles based on childhood maltreatment experiences (i.e., emotional abuse, emotional neglect, physical abuse, sexual abuse, physical neglect) and defenses (i.e., mature, mental inhibition and avoidance, and immature-depressive). A secondary aim was to analyze the associations between these profiles and impairment in personality functioning (i.e., self, interpersonal).

Based on the literature, we expected high maladaptive defenses (i.e., mental inhibition and avoidance, and immature-depressive) in childhood maltreatment profiles and high adaptive defenses (i.e., mature) in non-maltreated profiles. Second, we hypothesized that profiles characterized by maltreatment and maladaptive defenses would present higher impairments in self- and interpersonal functioning compared to non-maltreated and adaptive defenses profiles.

## Methods

### Participants

The inclusion criteria were: (a) aged 18–29 years, (b) living in Italy, and (c) fluent in the Italian language. To determine the minimum sample size required to detect small effects, an a-priori power analysis was conducted using the R package *semPower* (Moshagen, 2012). With alpha and RMSEA levels set to 0.05, the results indicated that a sample of *N* = 742 was needed to achieve 80% power to reject an incorrect model for 6 latent and 35 observed variables.

A non-clinical community sample of 1,453 cisgender emerging adults (*n* = 1078 assigned female at birth, 74.19%), aged 18–29 years (*M*_*age*_ = 24.36, *SD* = 2.79), was recruited. Among them, 75.98% (*n* = 1104) identified as heterosexual, while 8.53% (*n* = 124) identified as gay/lesbian, 11.49% (*n* = 167) as bisexual, and 3.99% (*n* = 58) as queer+. All participants resided in Italy and spoke fluent Italian; 96.90% (*n* = 1408) were Italian citizens. The majority (*n* = 792, 54.51%) were students, 28.77% (*n* = 418) were employed, 11.08% (*n* = 161) were both employed and a student, and 5.64% (*n* = 82) were unemployed. Regarding living arrangements, 63.66% (*n* = 925) lived with their parents, 17.34% (*n* = 252) lived alone, 10.87% (*n* = 158) lived with a partner, and 8.12% (*n* = 118) cohabited with friends.

### Procedure

Participants were recruited using snowball sampling techniques (e.g., posts on social media, word-of-mouth from participating emerging adults). Participation was contingent upon the acknowledgment and acceptance of a comprehensive informed consent form, which was presented prior to the survey on the Qualtrics platform. The voluntary nature of participation and stringent confidentiality measures were upheld and emphasized to participants. The survey was designed to prevent the identification of individual respondents, with response aggregation used to ensure data anonymity. Participants were informed that the entire assessment process would take approximately 20 min, and no incentives were provided for participation. The Territorial Ethics Committee of Lazio Area 2 (protocol n. 78.24 CET2 utv) approved the study.

### Measures

#### Childhood Maltreatment Experiences

Childhood maltreatment experiences were measured using the Italian version of the Childhood Trauma Questionnaire-Short Form (CTQ-SF; Bernstein et al., 2003; Sacchi et al., 2018), which comprises 28 items covering five domains: emotional abuse, physical abuse, sexual abuse, emotional neglect, and physical neglect. Sexual abuse (e.g., “Someone tried to touch me in a sexual way or tried to make me touch them”) involves any non-consensual sexual interaction initiated by an adult toward a minor; physical abuse (e.g., “Hit so hard that I had to see a doctor”) encompasses acts of violence resulting in physical harm requiring medical attention; emotional abuse (e.g., “My family said hurtful or insulting things to me”) involves verbal altercations that significantly impinge on a child’s psychological well-being; physical neglect (e.g., “Parents too drunk/high to take care”) is the failure to meet a child’s basic physiological needs; and emotional neglect (e.g., “Someone helped me feel important,” reverse-coded) involves the absence of necessary emotional and psychological support. Each item is rated on a 5-point Likert-scale ranging from 1 (*never*) to 5 (*very often*), with higher scores indicating greater maltreatment.

#### Defenses

Defenses were assessed using the Italian version of the Defense Mechanisms Rating Scales–Self-Report-30 (DMRS-SR-30; Di Giuseppe et al., 2020; Prout et al., 2022). The DMRS-SR-30 is a self-report questionnaire based on the Defense Mechanisms Rating Scale (Perry, 1990). Respondents rate items on a 5-point Likert scale ranging from 0 (*not at all*) to 4 (*very often/much*). The scale provides multiple levels of scoring, including an index of defensive maturity known as overall defensive functioning (ODF), different defensive levels organized hierarchically, and three defensive factors (i.e., mature, mental inhibition and avoidance, and immature-depressive). The *mature defense factor*, from the most to the least adaptive, includes affiliation, altruism, anticipation, humor, self-assertion, self-observation, sublimation, and suppression. The *mental inhibition and avoidance defense factor*, from the most to the least adaptive, includes isolation of affects, intellectualization, undoing, repression, dissociation, reaction formation, displacement, denial, and autistic fantasy. Finally, the immature-depressive defense factor, from the most to the least adaptive, includes idealization, devaluation, rationalization, projection, splitting of self-image, splitting of object-image, projective identification, passive aggression, help-rejecting complaining, and acting out.

#### Levels of Personality Functioning

Personality functioning was evaluated using the Italian version of the Levels of Personality Functioning Scale–Brief Form 2.0 (LPFS-BF 2.0; APA, 2013). The LPFS-BF follows the AMPD dual-domain structure, encompassing both self-functioning and interpersonal functioning (Hopwood et al., 2018). The self-functioning domain covers *identity*, which describes one’s sense of self and continuity (e.g., “I often do not know who I really am”), and *self-direction*, pertaining to one’s pursuit of coherent and meaningful short- and long-term life goals (e.g., “I have no sense of where I want to go in my life”). Interpersonal functioning encompasses *empathy*, describing one’s appreciation of others’ experiences and motivations (e.g., “I often do not fully understand why my behavior has a certain effect on others”), and *intimacy*, referring to the depth and quality of one’s close relationships (e.g., “I often feel very vulnerable when relations become more personal”). Items are rated on a 4-point Likert scale ranging from 1 (*completely untrue*) to 4 (*completely true*), with higher scores indicating greater impairment in the personality domain.

### Data Analysis

Data analyses were conducted using SPSS (version 24) and Mplus (version 8.4). First, the item pool was scrutinized for data entry accuracy and the presence of missing values. Subsequently, multivariate outliers were detected at the item level using Mahalanobis distance and an *χ*^*2*^ test with significance set to *p* <.001. The identified outliers were then removed from the sample. Next, normality assumptions were assessed by examining the skewness and kurtosis of each item, alongside the descriptive statistics.

An LPA was conducted to identify distinct profiles of emerging adults based on childhood maltreatment experiences and defenses. Since various forms of abuse often co-occur and may interact synergistically, analyzing them separately could overlook important patterns. An LPA approach addresses this issue by capturing underlying covariance structures among maltreatment experiences, allowing for the identification of meaningful subgroups based on shared patterns rather than treating individual maltreatment types as independent variables. Additionally, a profile-based approach is useful for operationalizing defenses, as the same defensive processes may manifest differently across distinct profiles.

Following the recommendations of Ferguson et al. (2020), the LPA process involved iterative model evaluation, assessment of model fit and interpretability, and exploration of profile patterns in the retained model. Model retention decisions were based on multiple fit indices, including the Bayesian Information Criterion (BIC), Sample-Adjusted BIC (SABIC), and Akaike’s Information Criterion (AIC), with lower values indicating superior model fit. The Lo-Mendell-Rubin (LMR) test was employed to compare each k-profile model to the k-1 model, with a non-significant result favoring the more parsimonious solution. Additionally, the bootstrap likelihood ratio test (BLRT) was used to determine whether each *k*-profile model provided a significantly better fit than a model with one fewer profile.

While entropy was assessed as a measure of classification certainty, it was not used as a primary model selection criterion given its limitations as a fit index. Models ranging from one to eight profiles were tested. Following model selection, a multivariate analysis of variance (MANOVA) was conducted to examine differences in personality functioning across latent profiles. Post hoc comparisons were adjusted using Bonferroni corrections to account for multiple comparisons (Tabachnick & Fidell, 2007). This person-centered approach provides a nuanced understanding of the complex relationship between childhood maltreatment experiences, defenses, and later personality functioning, examining how distinct trauma-defense profiles differentially influence self-functioning and interpersonal functioning in emerging adulthood.

## Results

### Missing Values Analysis and Descriptive Statistics

The dataset evidenced no missing values, enabling a comprehensive analysis. Additionally, 9.5% of the emerging adults (*n* = 138) were identified as multivariate outliers using Mahalanobis distance with *p* <.001. Following the removal of these outliers, the final sample comprised 1,315 participants (*M*_*age*_ = 24.33, *SD* = 2.75; 987 assigned female at birth, 75.06%; 1,005 identified as heterosexual, 76.43%). Variable normality was assessed using skewness and kurtosis indices, which ranged in distribution. The descriptive statistics indicated normal distribution characteristics for all variables except those related to sexual and physical abuse (see Table 1).

**Table 1:** Descriptives statistics of study variables (N = 1,315)

						Skewness		Kurtosis	
	*N*	Minimum	Maximum	*Mean*	*SD*	Statistic	Std. Error	Statistic	Std. Error
*Childhood maltreatment experiences*									
Emotional abuse	1,315	5.00	25.00	9.26	4.29	1.04	0.07	0.40	0.14
Physical abuse	1,315	5.00	20.00	5.87	1.98	3.23	0.07	12.13	0.14
Sexual abuse	1,315	5.00	23.00	5.28	1.31	6.53	0.07	52.93	0.14
Emotional neglect	1,315	5.00	25.00	9.89	3.79	0.74	0.07	0.10	0.14
Physical neglect	1,315	5.00	17.00	6.86	2.00	1.40	0.07	2.20	0.14
*Defenses*									
Mature	1,315	15.38	100.00	40.45	10.83	1.12	0.07	2.50	0.14
Mental inhibition/Avoidance	1,315	0.00	60.00	28.85	7.81	-0.28	0.07	0.65	0.14
Immature-Depressive	1,315	0.00	51.61	28.79	8.11	-0.62	0.07	0.81	0.14
*Personality functioning*									
Self-functioning	1,315	6.00	24.00	14.53	4.30	0.00	0.07	-0.62	0.14
Interpersonal functioning	1,315	6.00	23.00	12.01	3.28	0.23	0.07	-0.22	0.14

### LPA

As shown in Table 2, model fit indices supported a two-profile solution based on childhood maltreatment experiences and defenses. The Lo-Mendell-Rubin (LMR) test indicated a statistically significant improvement from one to two profiles (*p* =.000) but failed to reach significance for the transition from two to three profiles (*p* =.550), suggesting that additional profiles did not provide a meaningful gain in model fit. Entropy was acceptable (0.89), supporting adequate classification certainty. The smallest class in the two-profile solution constituted 26% of the sample, ensuring sufficient profile stability.

**Table 2:** Fit statistics different latent profiles obtained by the association of various childhood traumatic experiences and defense mechanisms (N = 1,315)

Model	Log Likelihood	AIC	BIC	SABIC	Entropy	Smallest Class %	LMR *p*-value	LMR Meaning	BLRT *p*-value	BLRT Meaning
1	-36558.71	73157.42	73261.05	73197.52	-	-	-	-	-	-
2	-28312.24	56674.48	56804.02	56724.60	0.89	26.00	0.000	2 > 1	0.000	2 > 1
3	-27599.23	55266.45	55442.63	55334.62	0.93	3.00	0.55	3 > 2	0.000	3 > 2
4	-27167.77	54421.54	54644.35	54507.76	0.93	2.00	0.12	4 > 3	0.000	4 > 3
5	-26789.09	53682.18	53951.63	53786.45	0.94	1.00	0.60	5 > 4	0.000	5 > 4
6	-26490.35	53102.70	53418.78	53225.01	0.91	1.00	0.01	6 > 5	0.000	6 > 5
7	-26246.09	52632.18	52994.89	52772.53	0.67	0.50	0.70	7 > 6	0.000	7 > 6
8	-259151.48	51988.30	52397.64	52146.70	0.92	0.001	0.25	8 > 7	0.000	8 > 7

Note. The Lo-Mendell Ruben (LMR) test and the bootstrap likelihood ratio test (BLRT) compared the current model to one model with *k*-1 profiles

Although AIC, BIC, and SABIC values continued to decrease with additional profiles, the magnitude of improvement was substantially larger between one and two profiles (ΔAIC = -16,482.95; ΔBIC = -16,457.04; ΔSABIC = -16,472.92) compared to subsequent model transitions (e.g., two to three profiles: ΔAIC = -1,408.03; ΔBIC = -1,361.39; ΔSABIC = -1,389.98). This pattern of diminishing returns suggested that the most meaningful differentiation occurred at the two-profile level, supporting its selection.

### Profile Characterization

**Fig. 1:**
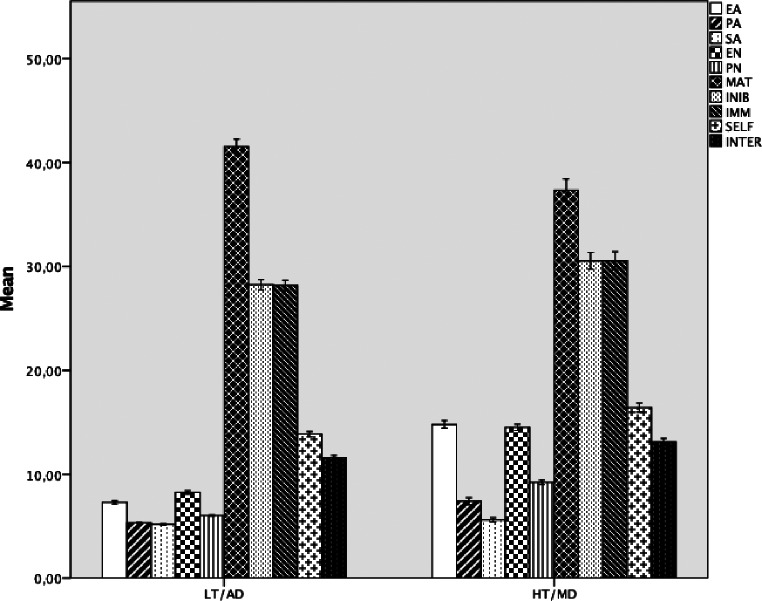
Mean differences in childhood maltreatment, defenses, and personality functioning across the two latent profiles (N = 1,315). Note. Error bars represent 95% confidence intervals. HT/MD = High-Trauma/Maladaptive Defenses profile; LT/AD = Low-Trauma/Adaptive Defenses profile. EA = Emotional Abuse, PA = Physical Abuse, SA = Sexual Abuse, EN = Emotional Neglect, PN = Physical Neglect, MAT = Mature Defenses, INIB = Mental Inhibition/Avoidance, IMM = Immature Depressive Defenses, SELF = Self-Functioning, INTER = Interpersonal Functioning

The first profile, labeled *High-Trauma/Maladaptive Defenses* (HT/MD; *n* = 971; 74.0%), was characterized by higher levels of emotional abuse (*M* = 14.81, *SD* = 3.57), physical abuse (*M* = 7.40, *SD* = 3.11), sexual abuse (*M* = 5.61, *SD* = 1.88), emotional neglect (*M* = 14.52, *SD* = 2.91), and physical neglect (*M* = 9.22, *SD* = 1.98) compared to the second profile (see Fig. 1).

Individuals in this profile exhibited greater reliance on maladaptive defenses, as reflected in lower mature defense scores (*M* = 37.34, *SD* = 10.24) and higher scores on mental inhibition/avoidance (*M* = 30.55, *SD* = 7.51) and immature depressive defenses (*M* = 30.55, *SD* = 8.01). These findings suggest that individuals exposed to greater childhood maltreatment are more likely to engage in defensive styles that hinder adaptive emotional processing and interpersonal functioning.

The second profile, labeled *Low-Trauma/Adaptive Defenses* (LT/AD; *n* = 344; 26.0%), included individuals with lower levels of emotional abuse (*M* = 7.30, *SD* = 2.39), physical abuse (*M* = 5.33, *SD* = 0.87), sexual abuse (*M* = 5.17, *SD* = 1.02), emotional neglect (*M* = 8.25, *SD* = 2.48), and physical neglect (*M* = 6.03, *SD* = 1.17). These individuals reported greater reliance on adaptive implicit emotion regulation strategies, reflected in higher mature defense scores (*M* = 41.55, *SD* = 10.83) and lower scores on mental inhibition/avoidance (*M* = 28.25, *SD* = 7.83) and immature depressive defenses (*M* = 28.16, *SD* = 8.06). Individuals in this profile also reported higher levels of self-functioning (*M* = 13.87, *SD* = 4.13) and interpersonal functioning (*M* = 11.61, *SD* = 3.20), suggesting an healthy personality functioning.

### Multivariate Analysis

A one-way multivariate analysis of variance (MANOVA) was conducted to examine differences in self-functioning, interpersonal functioning, childhood maltreatment, and defenses across the two latent profiles. Before conducting ANOVAs, assumptions of homogeneity of variance and covariance matrices were tested. Box’s M test for homogeneity of covariance matrices was statistically significant, Box’s M = 1692.87, *F*(55, 1440091.49) = 30.44, *p* <.001, indicating a violation of this assumption. Given this, Pillai’s Trace was used as the preferred multivariate statistic due to its robustness to heterogeneity of covariance matrices. Pillai’s Trace indicated a statistically significant difference between the two maltreatment-defense profiles on the combined dependent variables, *F*(10, 1304) = 298.01, *p* <.001; Pillai’s Trace = 0.696.

Then, Levene’s test of equality of error variances was also significant for childhood maltreatment variables, including emotional abuse, *F*(1, 1313) = 83.96, *p* <.001; physical abuse, *F*(1, 1313) = 717.24, *p* <.001; sexual abuse, *F*(1, 1313) = 95.36, *p* <.001; emotional neglect, *F*(1, 1313) = 7.97, *p* =.005; and physical neglect, *F*(1, 1313) = 123.22, *p* <.001, indicating heterogeneity of variance across groups. Given this, Welch’s ANOVA was used for these variables to account for unequal variances. For self-functioning, interpersonal functioning, and defense mechanisms, which met the homogeneity assumption, standard ANOVA was conducted. Welch’s ANOVA results indicated significant differences between profiles in emotional abuse, *Welch’s F*(1, 455.97) = 1310.08, *p* <.001; physical abuse, *Welch’s F*(1, 362.38) = 148.08, *p* <.001; sexual abuse, *Welch’s F*(1, 415.81) = 17.13, *p* <.001; emotional neglect, *Welch’s F*(1, 529.37) = 1269.84, *p* <.001; and physical neglect, *Welch’s F*(1, 430.30) = 792.29, *p* <.001.

For self-functioning, interpersonal functioning, and defenses, standard ANOVA results showed significant differences in self-functioning, *F*(1, 1313) = 95.38, *p* <.001; interpersonal functioning, *F*(1, 1313) = 54.95, *p* <.001; mature defenses, *F*(1, 1313) = 39.58, *p* <.001; mental inhibition/avoidance, *F*(1, 1313) = 22.40, *p* <.001; and immature depressive defenses, *F*(1, 1313) = 22.35, *p* <.001. Effect sizes, measured using Cohen’s *d*, indicated large differences between the two profiles in emotional abuse (*d* = 2.73), emotional neglect (*d* = 2.41), and physical neglect (*d* = 2.24), whereas a smaller effect was found for physical abuse (*d* = 1.18) and sexual abuse (*d* = 0.34). Furthermore, individuals in the LT/AD profile exhibited higher self-functioning (*M* = 13.87, *SD* = 4.13) and interpersonal functioning (*M* = 11.61, *SD* = 3.20) compared to those in the HT/MD profile (*M* = 16.41, *SD* = 4.20 and *M* = 13.11, *SD* = 3.25, respectively).

In conclusion, the HT/MD profile exhibited higher childhood maltreatment experiences, greater reliance on avoidance and immature depressive defenses, and lower self- and interpersonal functioning compared to the LT/AD profile, which demonstrated greater use of mature defenses and healthier personality functioning.

## Discussion

The current study examined distinct maltreatment-defenses profiles using LPA, identifying two groups within a large community sample of emerging adults: HT/MD and LT/AD. Additionally, the study explored how these profiles differed in terms of impairments in self- and interpersonal personality functioning. The results contribute to a developmental psychopathology perspective of child maltreatment (Cicchetti & Banny, 2014) providing a nuanced understanding of how childhood maltreatment and defenses interact to shape personality functioning in emerging adulthood. In this vein, the use of LPA offered a unique perspective on the relationship between maltreatment and defenses, emphasizing a person-centered approach. In contrast to previous studies (e.g., Ferrajão et al., 2023; Finzi-Dottan & Karu, 2006; Mahmoudvand et al., 2024; Musetti et al., 2023; Wang et al., 2021), which predominantly relied on variable-centered methods to examine these associations, LPA allowed for the identification of distinct subgroups, capturing the comorbidity and heterogeneity in maltreatment exposure and defensive functioning.

According to the first hypothesis, the LPA results supported a two-profile solution, with 74% of the sample belonging to HT/MD (characterized by high levels of all maltreatment experiences, high scores on mental inhibition/avoidance and immature depressive defenses, and low levels of immature defenses), and 26% of the sample falling into the LT/AD (characterized by low levels of all maltreatment experiences, low reliance on mental inhibition/avoidance and immature depressive defenses, and high levels of immature defenses). These findings align with previous evidence suggesting that childhood maltreatment is often related to maladaptive defensive (e.g., Ferrajão et al., 2023; Mahmoudvand et al., 2024). Furthermore, the higher levels of mature defenses in the LT/AD profile support the idea that, in the absence of severe trauma, individuals are more likely to develop adaptive defenses (Vaillant, 1977).

Similar to research on explicit emotion regulation (Morris et al., 2007), these results highlighted the critical role of early family environment in shaping individual defensive functioning (McWilliams, 2011). Childhood maltreatment is most perpetuated by parents or primary caregivers, and these experiences hinder the adaptive development of individual mental functioning across multiple domains, such as mentalization and defensive functioning (Lingiardi & McWilliams, 2017; Yang & Huang, 2024). Additionally, Cramer (2015a) suggested that immature defenses emerge in childhood, whereas mature defenses develop later, as they require more complex cognitive processes.

When multiple forms of maltreatment configure a repetitive pattern of caregiver–child interactions, as seen in self and other mental representations (Cicchetti & Valentino, 2006), the associated immature and neurotic defenses may become internalized and integrated into the personality structure, further hindering the emergence of more adaptive defenses. For example, in an effort to protect their stability when faced with an abusive caregiver, children may deny the abuse (i.e., denial), split the image of the abusive caregiver into “bad” and “good” (i.e., splitting), devalue themself as deserving of the abuse (i.e., devaluation of self). These defenses permit distorting the reality of maltreatment, ultimately maintaining a relationship with the abusive caregiver. As a result, early maltreatment experiences might hinder the development of adaptive defensive functioning, leading emerging adults to unconsciously rely on trauma-driven defenses when facing stressors (Cramer, 2015a).

Considering that previous studies have found that high maltreatment co-occurrence groups tend to be the least prevalent within community samples (Debowska et al., 2017), the high prevalence of the HT/MD profile (74%) in this study is unexpected. Compared with the LT/AD group, the HT/MD profile exhibited significantly higher levels of childhood maltreatment across all measured domains, with particularly elevated levels of emotional abuse, emotional neglect, and physical neglect. This pattern is consistent with meta-analytic evidence indicating that emotional abuse is the most frequently reported form of maltreatment (Stoltenborgh et al., 2015). Similarly, a recent umbrella review suggested that neglect may serve as the foundation for all forms of maltreatment, as an actively abusive parent is simultaneously failing to fulfill their caregiving role (Massullo et al., 2023). Furthermore, given that the sample was predominantly female, and that women tend to report higher levels of emotional abuse and neglect (Moody et al., 2018), it is possible that the high prevalence of the HT/MD profile is partly attributable to sample characteristics.

In line with our second hypothesis, the two identified profiles exhibited different levels of impairment in personality functioning. The HT/MD group reported greater impairments in both self- and interpersonal functioning compared with the LT/AD profile. These findings corroborate previous research showing the detrimental effect of childhood maltreatment on personality functioning (Bach et al., 2022; Freier et al., 2022; Gander et al., 2020; Krakau et al., 2021). Given the high prevalence of emotional abuse and neglect in HT/MD profile, severe impairments in personality functioning in this profile align with previous findings from studies utilizing the AMPD model (Bach et al., 2022; d’Huart et al., 2022; Gander et al., 2020) and the categorical and DSM-based approach to personality disorders (Cohen et al., 2013; Lobbestal et al., 2010), as well as meta-analytic findings on the effects of childhood maltreatment on dysfunctional personality traits or disorders (e.g., Porter et al., 2020).

Of note, family contexts involving psychological abuse and neglect often lack attuned and empathic caregiving, preventing children from developing the ability to mirror their own affective and cognitive states (Fonagy & Luyten, 2018). This deficit fosters perceptions of others as unavailable and incapable of understanding their feelings and thoughts, leading to expectations of humiliation, teasing, or being ignored in social interactions. Internalized criticism, humiliation, and feelings of inadequacy, stemming from early maltreatment experiences, may result in self-representations of unworthiness and unlovability, impacting identity and self-direction. Such perceptions may also hinder empathy and the development of deep and lasting relationships, causing the avoidance of intimacy to prevent social shame or further disregard in later ages (i.e., emerging adulthood). Thus, maladaptive personality functioning arising from maltreatment may deprive emerging adults of essential personal and interpersonal resources, potentially impeding their adaptive completion of salient developmental tasks (Cicchetti & Valentino, 2006).

Furthermore, considering that the HT/MD profile exhibited higher maladaptive defenses, according to previous meta-analytic results (e.g., Aldao et al., 2010), maladaptive emotion regulation constitutes a stronger risk factor for psychopathology than a lack of adaptive emotion regulation. Under these circumstances, the predominant use of maladaptive defenses developed within abusive and neglecting parent-child relationships may hinder healthy personality development, fostering negative representations of one’s self and others that will likely impair personality functioning (Kernberg, 2012; Vaillant, 1977).

Guided by a developmental psychopathology framework (Cicchetti & Banny, 2014), the present findings highlight the detrimental impact of various co-occurrence forms of childhood maltreatment on the domains of personality functioning in emerging adulthood. Maltreatment experiences often result in fragmented, negative, and contradictory self and caregiver representations. Although there is potential for change and resilience over the lifespan, these negative representations may persist and generalize to new social situations, leading emerging adults to repeat negative relational patterns (Toth et al., 1997).

These findings underscore the importance of considering both environmental (i.e., childhood maltreatment) and individual (i.e., defense mechanisms) factors when assessing personality impairment in emerging adulthood. Limited research has focused on the developmental challenges of emerging adulthood in the context of childhood maltreatment, including the development of a cohesive self-identity and close relationships (Cicchetti, 2016). As Sharp (2020) suggested, the AMPD model—and especially the related Criterion A—promotes an integrated developmental theory of personality, considering maturational shifts in identity, self-esteem, self-regulation, self-reflection, goal-setting, perspective-taking, comprehension of others, and the quality and duration of close relationships. Our findings also support an organizational perspective on development (Cicchetti & Valentino, 2006), suggesting that, within the context of maltreatment, poor resolution of earlier developmental issues (i.e., emotion regulation) may impair the adaptive development of self and other representations. Subsequently, this may disrupt the acquisition of competencies in emerging adulthood (i.e., identity formation and development of intimate relationships).

In summary, maladaptive self and interpersonal functioning in emerging adults may result from the interrelations of various contextual and individual factors. Following a cascading effect, the co-occurrence of maltreatment hinders the use of adaptive implicit emotion regulation strategies and increases the reliance on immature and neurotic defenses. The compounding effects of maltreatment and maladaptive defenses may significantly impair self-representation and hinder empathy and intimacy, thereby increasing the risk of overall personality dysfunction.

### Limitations

The present study has several limitations. First, although the large non-clinical sample size was a strength, the predominance of female participants limits the generalizability of the findings. Future research should include a more gender-balanced sample of emerging adults, also considering evidence that men and women experience different rates of each maltreatment type (e.g., sexual abuse tends to be higher among women, and physical violence more common among men; Australian Bureau of Statistics, 2017; Moody et al., 2018). Second, the cross-sectional design limited our ability to determine the long-term causal effects of childhood maltreatment on personality functioning.

Third, reliance on self-report measures may have introduced response bias and inflated associations between variables due to shared method variance. This is particularly relevant for personality functioning and defenses, as personality data from different rater types have been found to show significant but generally small to moderate correlations (Bradley et al., 2007). While the DMRS-SR-30 has demonstrated good criterion and concurrent validity, respectively, with the observer-rated instruments of DMRS and DMRS-Q sort in the Italian sample (Di Giuseppe et al., 2020), the self-reported nature of defensive functioning remains a limitation. Furthermore, retrospective reports of childhood adversities may be subject to recall bias. To address these issues, future studies should employ a longitudinal design, incorporating multi-method assessments such as clinical interviews or observer methods to assess childhood maltreatment, defensive styles, and personality functioning.

Fourth, while previous studies have found that LPFS has high predictive validity for maladaptive personality traits (Hopwood et al., 2018), we did not evaluate AMPD Criterion B. Future research should include both AMPD criteria, exploring the associations of childhood maltreatment with both personality functioning and pathological personality traits (e.g., negative affectivity, disinhibition). Finally, other unobserved variables, such as failure in the mentalization of trauma (Berthelot et al., 2022) and epistemic trust (Benzi et al., 2023; Cruciani et al., 2025), may play a role in the observed associations. Future studies should investigate these and other cognitive-affective processes as additional contributors to personality functioning impairments following maltreatment.

### Clinical Implications

The introduction of the Alternative Model for Personality Disorders (AMPD) in the DSM-5 (APA, 2013), as well as the growing attention given to AMPD Criterion A domains (i.e., identity, self-direction, empathy, intimacy), allowed for a more developmentally sensitive elaboration of maladaptive personality functioning over the life course (Sharp & Wall, 2021; Tackett & Sharp, 2014). This has several implications for the transitional and challenging stage of emerging adulthood. However, despite the well-established impact of childhood maltreatment on personality, no studies have examined how different forms of maltreatment interact with defenses to shape personality functioning specifically in emerging adulthood.

From a developmental psychopathology perspective (Cicchetti & Banny, 2014), several clinical implications may be proposed. Emerging adults face numerous developmental challenges, such as identity exploration, increased commitment in relationships, and the assumption of responsibilities in new social contexts (Arnett, 2000). The fulfilment of these developmental challenges is closely linked to their self- and other- representations, which are key aspects of personality functioning. Thus, greater insight into the impact of childhood maltreatment on these domains may help to inform tailored interventions aimed at mitigating long-term negative consequences and increasing the likelihood of adaptive development.

Furthermore, the co-occurrence of maltreatment forms represents a significant familial risk factor for overreliance on maladaptive defensive strategies in emerging adults, potentially contributing to greater impairment in self and interpersonal personality functioning. This underscores the importance of early detection and careful assessment of such adverse childhood experiences, which may exacerbate the challenges of this life stage, hindering emerging adults from achieving their aspirations and establishing a stable adult life.

In both research and clinical practice, greater attention should be given to implicit emotion regulation, in addition to explicit emotional strategies (Gyurak, 2011). Based on the current findings, clinical work with maltreated emerging adults would benefit from the inclusion of defensive functioning as a target for preventive interventions. Similarly, both preventive and clinical interventions may aid help-seeking emerging adults in processing past psychological maltreatment, developing emotional and cognitive awareness, improving their self-reflection, and channeling their traumatic effects into constructive behaviors, thereby increasing their sense of mastery over past abuse.

Finally, emerging adults often begin preparing for their future roles as parents long before they experience pregnancy or form a romantic relationship (Scharf & Mayseless, 2011). By equipping them with the tools to navigate future adult and parental responsibilities effectively, clinical interventions may disrupt the cycle of maltreatment, preventing distorted self and other representations from impacting parental identity and the caregiving system.

## Data Availability

Data are available from the corresponding author upon reasonable request.

## References

[CR1] Aldao, A., Nolen-Hoeksema, S., & Schweizer, S. (2010). Emotion-regulation strategies across psychopathology: A meta-analytic review. *Clinical Psychology Review*, *30*, 217–237. 10.1016/j.cpr.2009.11.004 20015584 10.1016/j.cpr.2009.11.004

[CR2] American Psychiatric Association (2013). *Diagnostic and Statistical Manual of Mental Disorders (5th ed., DSM–5)*. Author.

[CR3] Arnett, J. J. (2000). Emerging adulthood: A theory of development from the late teens through the twenties. *American Psychologist*, *55*(5), 469–480. 10.1037/0003-066X.55.5.469 10842426

[CR4] Australian Bureau of Statistics (2017). *Personal safety survey, Australia, 2016*. http://www.abs.gov.au/ausstats/abs@.nsf/mf/4906.0

[CR92] Bach, B., Bo, S., & Simonsen, E. (2022). Maladaptive personality traits may link childhood trauma history to current internalizing symptoms. *Scandinavian Journal of Psychology*, *63*(5), 468–475. 10.1111/sjop.12830 10.1111/sjop.12830 PMC9790355 35606936

[CR5] Back, S. N., Flechsenhar, A., Bertsch, K., & Zettl, M. (2021). Childhood traumatic experiences and dimensional models of personality disorder in DSM-5 and ICD-11: Opportunities and challenges. *Current Psychiatry Reports*, *23*, 60. 10.1007/s11920-021-01265-5 34279729 10.1007/s11920-021-01265-5 PMC8289775

[CR6] Back, S. N., Zettl, M., Bertsch, K., & Taubner, S. (2020). Personality functioning, maladaptive traits, and childhood trauma. *Psychotherapeut*, *65*, 374–382. 10.1007/s00278-020-00445-7

[CR7] Baldwin, J. R., Wang, B., Karwatowska, L., Schoeler, T., Tsaligopoulou, A., Munafò, M. R., & Pingault, J. B. (2023). Childhood maltreatment and mental health problems: A systematic review and meta-analysis of quasi-experimental studies. *American Journal of Psychiatry*, *180*(2), 117–126. 10.1176/appi.ajp.20220174 36628513 10.1176/appi.ajp.20220174 PMC7614155

[CR8] Bender, D. S., Morey, L. C., & Skodol, A. E. (2011). Toward a model for assessing level of personality functioning in DSM–5, part I: A review of theory and methods. *Journal of Personality Assessment*, *93*(4), 332–346. 10.1080/00223891.2011.583808 22804672 10.1080/00223891.2011.583808

[CR9] Benzi, I. M. A., Carone, N., Parolin, L., Martin-Gagnon, G., Ensink, K., & Fontana, A. (2023). Different epistemic stances for different traumatic experiences: Implications for mentalization. *Research in Psychotherapy: Psychopathology Process and Outcome*, *26*(3), 708. 10.4081/ripppo.2023.708 38156583 10.4081/ripppo.2023.708 PMC10772857

[CR91] Bernstein, D. P., Stein, J. A., Newcomb, M. D., Walker, E., Pogge, D., Ahluvalia, T., Stokes, J.,Handelsman, L., Medrano, M., Desmond, D., & Zule, W. (2003). Development and validation of a brief screening version of the Childhood Trauma Questionnaire. *Child Abuse & Neglect*, *27*(2), 169–190. 10.1016/S0145-2134(02)00541-0 10.1016/s0145-2134(02)00541-0 12615092

[CR10] Berthelot, N., Savard, C., Lemieux, R., Garon-Bissonnette, J., Ensink, K., & Godbout, N. (2022). Development and validation of a self-report measure assessing failures in the mentalization of trauma and adverse relationships. *Child Abuse & Neglect*, *128*, 105017. 10.1016/j.chiabu.2021.105017 33692012 10.1016/j.chiabu.2021.105017

[CR11] Bradley, R., Hilsenroth, M., Guarnaccia, C., & Westen, D. (2007). Relationship between clinician assessment and self-assessment of personality disorders using the SWAP-200 and PAI. *Psychological Assessment*, *19*(2), 225–229. 10.1037/1040-3590.19.2.225 17563203 10.1037/1040-3590.19.2.225

[CR12] Callahan, K. L., & Hilsenroth, M. J. (2005). Childhood sexual abuse and adult defensive functioning. *Journal of Nervous & Mental Disease*, *193*(7), 473–479. 10.1097/01.nmd.0000168237.26124.47 15985842 10.1097/01.nmd.0000168237.26124.47

[CR13] Carone, N., Benzi, I. M. A., Muzi, L., Parolin, L. A. L., & Fontana, A. (2023). Problematic internet use in emerging adulthood to escape from maternal helicopter parenting: Defensive functioning as a mediating mechanism. *Research in Psychotherapy: Psychopathology Process and Outcome*, *26*(3), 693. 10.4081/ripppo.2023.693 37946531 10.4081/ripppo.2023.693 PMC10715189

[CR14] Carone, N., & Tracchegiani, J. (2025). Childhood maltreatment in maternal helpless caregiving: The mediating role of defensive functioning. *Journal of Family Trauma, Child Custody & Child Development*,*22*(1), 157–178. 10.1080/26904586.2024.2434855

[CR15] Caspi, A., Houts, R. M., Ambler, A., Danese, A., Elliott, M. L., Hariri, A., Harrington, H., Hogan, S., Poulton, R., Ramrakha, S., Rasmussen, L. J. H., Reuben, A., Richmond-Rakerd, L., Sugden, K., Wertz, J., Williams, B. S., & Moffitt, T. E. (2020). Longitudinal assessment of mental health disorders and comorbidities across 4 decades among participants in the Dunedin birth cohort study. *JAMA Network Open*, *3*(4), e203221. 10.1001/jamanetworkopen.2020.3221 32315069 10.1001/jamanetworkopen.2020.3221 PMC7175086

[CR16] Chanen, A. M., & Thompson, K. N. (2019). The age of onset of personality disorders. In de G. Girolamo, P. McGorry, & N. Sartorius (Eds.), *Age of onset of mental disorders*. Springer. 10.1007/978-3-319-72619-9_10

[CR17] Cicchetti, D. (2016). Socioemotional, personality, and biological development: Illustrations from a multilevel developmental psychopathology perspective on child maltreatment. *Annual Review of Psychology*, *67*, 187–211. 10.1146/annurev-psych-122414-033259 26726964 10.1146/annurev-psych-122414-033259

[CR18] Cicchetti, D., & Banny, A. (2014). A developmental psychopathology perspective on child maltreatment. In M. Lewis & K. D. Rudolph (Eds.), *Handbook of developmental psychopathology* (3rd ed., pp. 723–741). Springer. 10.1007/978-1-4614-9608-3_37

[CR19] Cicchetti, D., & Valentino, K. (2006). An ecological-transactional perspective on child maltreatment: Failure of the average expectable environment and its influence on child development. In D. Cicchetti, & D. J. Cohen (Eds.), *Developmental psychopathology: Risk, disorder, and adaptation* (2nd ed., pp. 129–201). John Wiley & Sons, Inc. 10.1002/9780470939406.ch4

[CR20] Clarkin, J. F., Caligor, E., & Sowislo, J. F. (2020). An object relations model perspective on the alternative model for personality disorders (DSM-5). *Psychopathology*, *53*(3–4), 141–148. 10.1159/000508353 32698184 10.1159/000508353 PMC7949219

[CR21] Cohen, L. J., Foster, M., Nesci, C., Tanis, T., Halmi, W., & Galynker, I. (2013). How do different types of childhood maltreatment relate to adult personality pathology? *Journal of Nervous and Mental Disease*, *201*(3), 234–243. 10.1097/NMD.0b013e3182848ac4 23417013 10.1097/NMD.0b013e3182848ac4

[CR23] Cramer, P. (1999). Personality, personality disorders, and defense mechanisms. *Journal of Personality*, *67*(3), 535–554. 10.1111/1467-6494.00064 10483120 10.1111/1467-6494.00064

[CR24] Cramer, P. (2015a). Defense mechanisms: 40 years of empirical research. *Journal of Personality Assessment*, *97*(2), 114–122. 10.1080/00223891.2014.947997 25157632 10.1080/00223891.2014.947997

[CR22] Cramer, P. (2015b). Understanding defense mechanisms. *Psychodynamic Psychiatry*, *43*(4), 523–552. 10.1521/pdps.2015.43.4.523 26583439 10.1521/pdps.2015.43.4.523

[CR25] Cramer, P., & Block, J. (1998). Preschool antecedents of defense mechanism use in young adults: A longitudinal study. *Journal of Personality and Social Psychology*, *74*(1), 159–169. 10.1037//0022-3514.74.1.159 10.1037//0022-3514.74.1.159 9457780

[CR93] Cruciani, G., Fontana, A., Benzi, I. M. A., Cacioppo, M., Muzi, L., Parolin, L., Tracchegiani, J., & Carone, N. (2025). Defensive levels in narcissistic profiles: Associations with epistemic trust, mistrust, and credulity in emerging adulthood. *Current Psychology*. 10.1007/s12144-025-07850-8

[CR27] Debowska, A., Willmott, D., Boduszek, D., & Jones, A. D. (2017). What do we know about child abuse and neglect patterns of co-occurrence? A systematic review of profiling studies and recommendations for future research. *Child Abuse & Neglect*, *70*, 100–111. 10.1016/j.chiabu.2017.06.014 28609690 10.1016/j.chiabu.2017.06.014

[CR28] de Heer, C., Bi, S., Finkenauer, C., Alink, L., & Maes, M. (2024). The association between child maltreatment and loneliness across the lifespan: A systematic review and multilevel meta-analysis. *Child Maltreatment*, *29*(2), 388–404. 10.1177/10775595221103420 35652822 10.1177/10775595221103420 PMC11539460

[CR26] d’Huart, D., Hutsebaut, J., Seker, S., Schmid, M., Schmeck, K., Bürgin, D., & Boonmann, C. (2022). Personality functioning and the pathogenic effect of childhood maltreatment in a high-risk sample. *Child and Adolescent Psychiatry and Mental Health*, *16*(1). 10.1186/s13034-022-00527-1 10.1186/s13034-022-00527-1 PMC9710065 36451183

[CR29] Di Giuseppe, M., Perry, J. C., Lucchesi, M., Michelini, M., Vitiello, S., Piantanida, A., Fabiani, M., Maffei, S., & Conversano, C. (2020). Preliminary reliability and validity of the DMRS-SR-30, novel self-report measure based on the Defense Mechanisms Rating Scales. *Frontiers in Psychiatry*, *11*, 870. 10.3389/fpsyt.2020.00870 33005160 10.3389/fpsyt.2020.00870 PMC7479239

[CR30] Dong, M., Anda, R. F., Felitti, V. J., Dube, S. R., Williamson, D. F., Thompson, T. J., Loo, C. M., & Giles, W. H. (2004). The interrelatedness of multiple forms of childhood abuse, neglect, and household dysfunction. *Child Abuse & Neglect*, *28*(7), 771–784. 10.1016/j.chiabu.2004.01.008 15261471 10.1016/j.chiabu.2004.01.008

[CR31] Doyle, C., & Cicchetti, D. (2017). From the cradle to the grave: The effect of adverse caregiving environments on attachment and relationships throughout the lifespan. *Clinical Psychology: Science and Practice*, *24*(2), 203–217. 10.1111/cpsp.12192 28924334 10.1111/cpsp.12192 PMC5600283

[CR32] Ferguson, S. L., Moore, E. W. G., & Hull, D. M. (2020). Finding latent groups in observed data: A primer on latent profile analysis in Mplus for applied researchers. *International Journal of Behavioral Development*, *44*(5), 458–468. 10.1177/0165025419881721

[CR33] Ferrajão, P., Batista, C. I., & Elklit, A. (2023). Polytraumatization, defense mechanisms, PTSD and complex PTSD in Indian adolescents: A mediation model. *BMC Psychology*, *11*, 411. 10.1186/s40359-023-01456-0 38001536 10.1186/s40359-023-01456-0 PMC10675876

[CR35] Finzi-Dottan, R., & Karu, T. (2006). From emotional abuse in childhood to psychopathology in adulthood. *Journal of Nervous & Mental Disease*, *194*(8), 616–621. 10.1097/01.nmd.0000230654.49933.23 16909071 10.1097/01.nmd.0000230654.49933.23

[CR34] Finzi, R., Har-Even, D., & Weizman, A. (2003). Comparison of ego defenses among physically abused children, neglected, and non-maltreated children. *Comprehensive Psychiatry*, *44*(5), 388–395. 10.1016/s0010-440x(03)00106-8 14505299 10.1016/S0010-440X(03)00106-8

[CR36] Fonagy, P., & Luyten, P. (2018). Attachment, mentalizing, and the self. In W. J. Livesley, & R. Larstone (Eds.), *Handbook of personality disorders: Theory, research, and treatment* (2nd ed., pp. 123–140). The Guilford Press.

[CR37] Freier, A., Kruse, J., Schmalbach, B., Zara, S., Werner, S., Brähler, E., Fegert, J. M., & Kampling, H. (2022). The mediation effect of personality functioning between different types of child maltreatment and the development of depression/anxiety symptoms– A German representative study. *Journal of Affective Disorders*, *299*, 408–415. 10.1016/j.jad.2021.12.020 34906643 10.1016/j.jad.2021.12.020

[CR38] Gander, M., Buchheim, A., Bock, A., Steppan, M., Sevecke, K., & Goth, K. (2020). Unresolved attachment mediates the relationship between childhood trauma and impaired personality functioning in adolescence. *Journal of Personality Disorders*, *34*(Supplement B), 84–103. 10.1521/pedi_2020_34_468 31990614 10.1521/pedi_2020_34_468

[CR39] Gyurak, A., Gross, J. J., & Etkin, A. (2011). Explicit and implicit emotion regulation: A dual-process framework. *Cognition & Emotion*, *25*(3), 400–412. 10.1080/02699931.2010.544160 21432682 10.1080/02699931.2010.544160 PMC3280343

[CR40] Handley, E. D., Russotti, J., Rogosch, F. A., & Cicchetti, D. (2019). Developmental cascades from child maltreatment to negative friend and romantic interactions in emerging adulthood. *Development and Psychopathology*, *31*(5), 1649–1659. 10.1017/s095457941900124x 31718734 10.1017/S095457941900124X PMC6913033

[CR41] Hecht, K. F., Cicchetti, D., Rogosch, F. A., & Crick, N. R. (2014). Borderline personality features in childhood: The role of subtype, developmental timing, and chronicity of child maltreatment. *Development and Psychopathology*, *26*(3), 805–815. 10.1017/s0954579414000406 25047300 10.1017/S0954579414000406 PMC4141853

[CR42] Hopwood, C. J., Good, E. W., & Morey, L. C. (2018). Validity of the DSM–5 Levels of Personality Functioning Scale–Self report. *Journal of Personality Assessment*, *100*(6), 650–659. 10.1080/00223891.2017.1420660 29424568 10.1080/00223891.2017.1420660

[CR43] Iannattone, S., Schuiringa, H. D., Aleva, A., Koster, N., van Aken, M. A. G., Hessels, C. J., van der Heijden, P. T., & Laceulle, O. M. (2024). Unravelling the longitudinal relations between developmental milestones, general psychopathology, and personality functioning in a youth clinical sample. *Journal of Youth and Adolescence*. 10.1007/s10964-024-01971-2. Advance online publication. 38499819 10.1007/s10964-024-01971-2 PMC11226502

[CR44] Kampe, L., Zimmermann, J., Bender, D., Caligor, E., Borowski, A. L., Ehrenthal, J. C., Benecke, C., & Hörz-Sagstetter, S. (2018). Comparison of the structured DSM–5 clinical interview for the Level of Personality Functioning Scale with the Structured Interview of Personality Organization. *Journal of Personality Assessment*, *100*(6), 642–649. 10.1080/00223891.2018.1489257 30907713 10.1080/00223891.2018.1489257

[CR45] Kernberg, O. F. (1984). *Severe personality disorders: Psychotherapeutic strategies*. Yale University Press.

[CR46] Kernberg, O. F. (2012). Overview and critique of the classification of personality disorders proposed for DSM-V. *Schweizer Archiv Für Neurologie Und Psychiatrie*, *163*(7), 234–238. 10.4414/sanp.2012.00110

[CR47] Klein, E. M., Benecke, C., Kasinger, C., Brähler, E., Ehrenthal, J. C., Strauß, B., & Ernst, M. (2022). Eating disorder psychopathology: The role of attachment anxiety, attachment avoidance, and personality functioning. *Journal of Psychosomatic Research*, *160*, 110975. 10.1016/j.jpsychores.2022.110975 35763941 10.1016/j.jpsychores.2022.110975

[CR49] Leeb, R. T., Paulozzi, L. J., Melanson, C., Simon, T. R., & Arias, I. (2008). *Child maltreatment surveillance: Uniform definitions for public health and recommended data elements, version 1.0*. Centers for Disease Control and Prevention, National Center for Injury Prevention and Control. 10.1037/e587022010-001

[CR50] Lingiardi, V., & McWilliams, N. (Eds.). (2017). *Psychodynamic Diagnostic Manual: PDM-2* (2nd ed.). The Guilford Press. 10.1002/wps.20233 PMC4471982 26043343

[CR51] Lobbestael, J., Arntz, A., & Bernstein, D. P. (2010). Disentangling the relationship between different types of childhood maltreatment and personality disorders. *Journal of Personality Disorders*, *24*(3), 285–295. 10.1521/pedi.2010.24.3.285 20545495 10.1521/pedi.2010.24.3.285

[CR52] Mahmoudvand, M., Zahrakar, K., & Hasani, J. (2024). Explaining the causal relationships between childhood maltreatment and attachment styles with forgiveness in betrayal victims: The mediating role of defensive mechanisms. *Journal of Assessment and Research in Applied Counseling*, *6*(1), 1–14. 10.61838/kman.jarac.6.1.1

[CR54] Massullo, C., De Rossi, E., Carbone, G. A., Imperatori, C., Ardito, R. B., Adenzato, M., & Farina, B. (2023). Child maltreatment, abuse, and neglect: An umbrella review of their prevalence and definitions. *Clinical Neuropsychiatry*, *20*(2), 72–99. 10.36131/cnfioritieditore20230201 37250758 10.36131/cnfioritieditore20230201 PMC10211430

[CR53] McWilliams, N. (2011). *Psychoanalytic diagnosis: Understanding personality structure in the clinical process*. The Guilford Press.

[CR55] Møller, L., Meisner, M. W., Søgaard, U., Elklit, A., & Simonsen, E. (2021). Assessment of personality functioning in ICD-11 Posttraumatic Stress Disorder and Complex Posttraumatic Stress Disorder. *Personality Disorders*, *12*(5), 466–474. 10.1037/per0000491 34435806 10.1037/per0000491

[CR56] Moody, G., Cannings-John, R., Hood, K., Kemp, A., & Robling, M. (2018). Establishing the international prevalence of self-reported child maltreatment: A systematic review by maltreatment type and gender. *BMC Public Health*, *18*, 1164. 10.1186/s12889-018-6044-y 30305071 10.1186/s12889-018-6044-y PMC6180456

[CR57] Morris, A. S., Silk, J. S., Steinberg, L., Myers, S. S., & Robinson, L. R. (2007). The role of the family context in the development of emotion regulation. *Social Development*, *16*(2), 361–388. 10.1111/j.1467-9507.2007.00389.x 19756175 10.1111/j.1467-9507.2007.00389.x PMC2743505

[CR58] Moshagen, M. (2012). The model size effect in SEM: Inflated goodness-of-fit statistics are due to the size of the covariance matrix. *Structural Equation Modeling: A Multidisciplinary Journal*, *19*(1), 86–98. 10.1080/10705511.2012.634724

[CR59] Musetti, A., Gagliardini, G., Lenzo, V., & Cella, S. (2023). From childhood emotional maltreatment to disordered eating: A path analysis. *Psychoanalytic Psychology*, *40*(2), 90–98. 10.1037/pap0000438

[CR60] Perry, J. C. (1990). *Defense Mechanism Rating Scales* (DMRS) (5th ed.). Author. 10.1002/jclp.22089 24706519

[CR61] Perry, J. C., Presniak, M. D., & Olson, T. R. (2013). Defense mechanisms in schizotypal, borderline, antisocial, and narcissistic personality disorders. *Psychiatry: Interpersonal and Biological Processes*, *76*(1), 32–52. 10.1521/psyc.2013.76.1.32 10.1521/psyc.2013.76.1.32 23458114

[CR62] Persike, M., Seiffge-Krenke, I., Cok, F., Głogowska, K., Pavlopoulos, V., Tantaros, S., Perchec, C., Rohail, I., & Saravia, J. C. (2020). Emerging adults’ psychopathology in seven countries: The impact of identity-related risk factors. *Emerging Adulthood*, *8*(3), 179–194. 10.1177/2167696818791108

[CR63] Porter, C., Palmier-Claus, J., Branitsky, A., Mansell, W., Warwick, H., & Varese, F. (2020). Childhood adversity and borderline personality disorder: A meta-analysis. *Acta Psychiatrica Scandinavica*, *141*, 6–20. 10.1111/acps.13118 31630389 10.1111/acps.13118

[CR64] Prout, T. A., Di Giuseppe, M., Zilcha-Mano, S., Perry, J. C., & Conversano, C. (2022). Psychometric properties of the Defense Mechanisms Rating Scales-Self-Report-30 (DMRS-SR-30): Internal consistency, validity and factor structure. *Journal of Personality Assessment*, *104*(6), 833–843. 10.1080/00223891.2021.2019053 35180013 10.1080/00223891.2021.2019053

[CR65] Prunas, A., Di Pierro, R., Huemer, J., & Tagini, A. (2019). Defense mechanisms, remembered parental caregiving, and adult attachment style. *Psychoanalytic Psychology*, *36*(1), 64–72. 10.1037/pap0000158

[CR48] Krakau, L., Tibubos, A. N., Beutel, M. E., Ehrenthal, J. C., Gieler, U., & Brähler, E. (2021). Personality functioning as a mediator of adult mental health following child maltreatment. *Journal of Affective Disorders*, *291*, 126–134. 10.1016/j.jad.2021.05.006 34034217 10.1016/j.jad.2021.05.006

[CR66] Roche, M. J., Jacobson, N. C., & Phillips, J. J. (2018). Expanding the validity of the Level of Personality Functioning Scale observer report and self-report versions across psychodynamic and interpersonal paradigms. *Journal of Personality Assessment*, *100*(6), 571–580. 10.1080/00223891.2018.1475394 29897794 10.1080/00223891.2018.1475394

[CR68] Sacchi, C., Vieno, A., & Simonelli, A. (2018). Italian validation of the Childhood Trauma Questionnaire—Short Form on a college group. *Psychological Trauma: Theory Research Practice and Policy*, *10*(5), 563–571. 10.1037/tra0000333 29016156 10.1037/tra0000333

[CR67] Sarrar, L., & Goth, K. (2022). Defense mechanisms reloaded in the light of impaired personality functioning: An attempt of clarification and simplification resulting in the DSQ-22-A for adolescents. *Frontiers in Psychiatry*, *13*, 866837. 10.3389/fpsyt.2022.866837 35722566 10.3389/fpsyt.2022.866837 PMC9198968

[CR69] Scharf, M., & Mayseless, O. (2011). Buds of parenting in emerging adult males: What we learned from our parents. *Journal of Adolescent Research*, *26*(4), 479–505. 10.1177/0743558411402339

[CR70] Sharp, C. (2020). Adolescent personality pathology and the Alternative Model for Personality Disorders: Self development as nexus. *Psychopathology*, *53*(3–4), 198–204. 10.1159/000507588 32464626 10.1159/000507588

[CR71] Sharp, C., & Wall, K. (2021). DSM-5 level of personality functioning: Refocusing personality disorder on what it means to be human. *Annual Review of Clinical Psychology*, *17*(1), 313–337. 10.1146/annurev-clinpsy-081219-105402 33306924 10.1146/annurev-clinpsy-081219-105402

[CR72] Skodol, A. E., Morey, L. C., Bender, D. S., & Oldham, J. M. (2015). The Alternative DSM-5 Model for Personality Disorders: A clinical application. *The American Journal of Psychiatry*, *172*(7), 606–613. 10.1176/appi.ajp.2015.14101220 26130200 10.1176/appi.ajp.2015.14101220

[CR73] Solmi, M., Radua, J., Olivola, M., Croce, E., Soardo, L., Salazar de Pablo, G., Il Shin, J., Kirkbride, J. B., Jones, P., Kim, J. H., Kim, J. Y., Carvalho, A. F., Seeman, M. V., Correll, C. U., & Fusar-Poli, P. (2021). Age at onset of mental disorders worldwide: Large-scale meta-analysis of 192 epidemiological studies. *Molecular Psychiatry*, *27*(1), 281–295. 10.1038/s41380-021-01161-7 34079068 10.1038/s41380-021-01161-7 PMC8960395

[CR75] Stoltenborgh, M., Bakermans-Kranenburg, M. J., Alink, L. R. A., & van IJzendoorn, M. H. (2015). The prevalence of child maltreatment across the Globe: Review of a series of meta‐analyses. *Child Abuse Review*, *24*(1), 37–50. 10.1002/car.2353

[CR74] Stone, L. E., Hurd, J. A., & Segal, D. L. (2023). The alternative model of personality disorders, trauma, and aging: Relationships with post-traumatic stress symptoms and the effect of cumulative trauma exposure. *Psychiatry Research Communications*, *3*(2). 10.1016/j.psycom.2023.100106

[CR76] Tabachnick, B. G., & Fidell, L. S. (2007). *Using multivariate statistics* (5th ed.). Allyn & Bacon/Pearson Education.

[CR77] Tackett, J. L., & Sharp, C. (2014). A developmental psychopathology perspective on personality disorder: Introduction to the special issue. *Journal of Personality Disorders*, *28*(1), 1–4. 10.1521/pedi.2014.28.1.1 24344882 10.1521/pedi.2014.28.1.1

[CR78] Toth, S. L., Cicchetti, D., Macfie, J., & Emde, R. N. (1997). Representations of self and other in the narratives of neglected, physically abused, and sexually abused preschoolers. *Development and Psychopathology*, *9*(4), 781–796. 10.1017/s0954579497001430 9449005 10.1017/s0954579497001430

[CR88] UNICEF (2024). *Nearly 400 million young children worldwide regularly experience violent discipline at home*. Retrieved from https://www.unicef.org/press-releases/nearly-400-million-young-children-worldwide-regularly-experience-violent-discipline

[CR80] Vaillant, G. E. (1977). *Adaptation to life*. Little.

[CR79] Vaillant, G. E. (2003). Mental health. *The American Journal of Psychiatry*, *160*(8), 1373–1384. 10.1176/appi.ajp.160.8.137 12900295 10.1176/appi.ajp.160.8.1373

[CR81] Vaillant, G. E. (2020). Defense mechanisms. In Zeigler-Hill, V., Shackelford, T.K. (Eds.) *Encyclopedia of personality and individual differences* (pp. 1024–1033). Springer. 10.1007/978-3-319-24612-3_1372

[CR82] Vittengl, J. R., Jarrett, R. B., Ro, E., & Clark, L. A. (2023). How can the DSM-5 Alternative Model of Personality Disorders advance understanding of depression? *Journal of Affective Disorders*, *320*, 254–262. 09.146. 10.1016/j.jad.2022 36191644 10.1016/j.jad.2022.09.146

[CR83] Wang, L., Yin, Y., Bian, Q., Zhou, Y., Huang, J., Zhang, P., Chen, S., Fan, H., Cui, Y., Luo, X., Tan, S., Wang, Z., Li, C. R., Tian, B., Tian, L., Hong, L. E., & Tan, Y. (2021). Immature defense mechanisms mediate the relationship between childhood trauma and onset of bipolar disorder. *Journal of Affective Disorders*, *278*, 672–677. 10.1016/j.jad.2020.10.029 33125910 10.1016/j.jad.2020.10.029

[CR84] Warmingham, J. M., Duprey, E. B., Handley, E. D., Rogosch, F. A., & Cicchetti, D. (2022). Patterns of childhood maltreatment predict emotion processing and regulation in emerging adulthood. *Development and Psychopathology*, *35*(2), 766–781. 10.1017/s0954579422000025 35287777 10.1017/S0954579422000025 PMC9474738

[CR85] Wright, A. G., Pincus, A. L., & Lenzenweger, M. F. (2011). Development of personality and the remission and onset of personality pathology. *Journal of Personality and Social Psychology*, *101*(6), 1351–1358. 10.1037/a0025557 21967009 10.1037/a0025557 PMC3222717

[CR86] Yalch, M. M. (2020). Psychodynamic underpinnings of the DSM–5 Alternative Model for Personality Disorder. *Psychoanalytic Psychology*, *37*(3), 219–231. 10.1037/pap0000262

[CR87] Yang, L., & Huang, M. (2024). Childhood maltreatment and mentalizing capacity: A meta-analysis. *Child Abuse & Neglect*, *149*, 106623. 10.1016/j.chiabu.2023.106623 38245975 10.1016/j.chiabu.2023.106623

[CR89] Zhang, H., Gao, X., Liang, Y., Yao, Q., & Wei, Q. (2023). Does child maltreatment reduce or increase empathy? A systematic review and meta-analysis. *Trauma Violence & Abuse*, *25*(1), 166–182. 10.1177/15248380221145734 10.1177/15248380221145734 36738112

[CR90] Zhang, H., Wang, W., Liu, S., Feng, Y., & Wei, Q. (2022). A meta-analytic review of the impact of child maltreatment on self-esteem: 1981 to 2021. *Trauma Violence & Abuse*, *24*(5), 3398–3411. 10.1177/15248380221129587 10.1177/15248380221129587 36341581

